# Capturing the continuum in biopsychosocial research: Measurement challenges within transdiagnostic and dimensional approaches to mental disorders

**DOI:** 10.1016/j.nsa.2025.105508

**Published:** 2025-02-08

**Authors:** Anna Schulze, Stefanie Lis

**Affiliations:** a Department of Clinical Psychology, Central Institute of Mental Health, Medical Faculty Mannheim, Heidelberg University, Germany; b Department of Psychosomatic Medicine and Psychotherapy, Central Institute of Mental Health, Medical Faculty Mannheim, Heidelberg University, Mannheim, Germany

Transdiagnostic and dimensional approaches are increasingly credible competitors of the categorical approach to diagnoses, posing new challenges when studying mechanisms of mental disorders. In the framework of the categorical approach, we investigate changes in biopsychosocial processes within distinct categories of mental disorders such as schizophrenia, depression or personality disorders like Borderline Personality Disorder, often in comparison with forms of healthy functioning. In contrast, transdiagnostic and dimensional approaches aim to capture the association of changes in biopsychosocial processes with the frequency or intensity of attributes of continuous dimensions across individuals and/or diagnostic categories, understanding a dimension as a continuous variable resulting from summing or averaging indicators of the same construct (Hopwood et al., 2023). The dimensions may encompass behaviours, such as disruptive behaviours, symptoms such as fear, or traits of personality structure. In any case, the attributes regarded as indicative for a specific dimension are assumed (i) to vary among individuals; and (ii) to be relevant to the whole population to differing degrees. Accordingly, measurement instruments need to accurately capture the attribute of interest across its whole continuum with comparable validity and reliability. While the need for new measurement tools has already been emphasised (e.g., (Monaghan and Bizumic, 2023)), we still face the problem that studies to date have often used self-report questionnaires developed to quantify attributes in either non-clinical or specific clinical populations. As a result, measurement instruments may differ in how well they differentiate between individuals on different sectors of the continuum.

One example is the measurement of attributes associated with the Borderline personality phenotype. In biopsychosocial research, two different measurement instruments have been well-established: the Borderline subscale of the Personality Assessment Inventory (PAI-BOR), which measures Borderline personality features most often used in non-clinical samples (Morey, 1991); and the Borderline Symptom List (BSL-23) most often applied to measure Borderline Personality Disorder symptoms in clinical samples (Kleindienst et al., 2020). Most studies use one of these instruments. We analysed the association between both assessments in patients diagnosed with Borderline Personality Disorder and healthy individuals recruited as a non-clinical control group with pooled data from different studies conducted at the Central Institute of Mental Health.

Both instruments show variation across the full spectrum of possible scores (Fig. 1). They differentiate well between samples of healthy control participants and patients diagnosed with Borderline Personality Disorder, suggesting that both instruments are well-suited to categorise individuals in the context of a categorical approach (in this sample specificity: PAI-BOR: .99, BSL-23: .98; sensitivity: PAI-BOR: .87, BSL-23: .76). Their scores are highly correlated (N = 305, r = .84, 95%CI: .81-.87, p < .001) and show high reliability estimates (Cronbach-*α*, PAI-BOR: .95, 95%CI: .94-.96, BSL-23: .98, 95%CI: .97-.98) suggesting that each may be well-suited to capture the attribute of interest within the context of a dimensional approach. However, a closer look reveals that both instruments function very differently when capturing the Borderline personality phenotype across the continuum of healthy control and individuals diagnosed with BPD. Our data show that both scales miss a large portion of inter-individual variance in either the non-clinical or the clinical sample, respectively. In line, the shared variance between both instruments drops to 28.6% for those with lower scores (healthy control group) and 32.7% for those with higher scores (patients with Borderline Personality Disorder). This may not be surprising given that the PAI-BOR aims to measure more stable personality features, whereas the BSL targets Borderline Personality Disorder symptoms, which vary across the course of the disorder. Nevertheless, differences between the two instruments may have serious consequences for the investigation of mechanisms within the framework of a dimensional approach: correlations between the measure of the dimensional attribute and the quantity or quality of biopsychosocial alterations may differ depending on the instrument used, potentially leading to conflicting interpretations of altered biopsychosocial mechanisms. While the problem that different instruments do not always align is well known, disentangling the association of borderline personality features and Borderline personality disorder symptoms with changes in biopsychosocial processes might increase our understanding of etiological factors and tailoring interventions for those in need of biopsychosocial interventions.

**Fig. 1:**
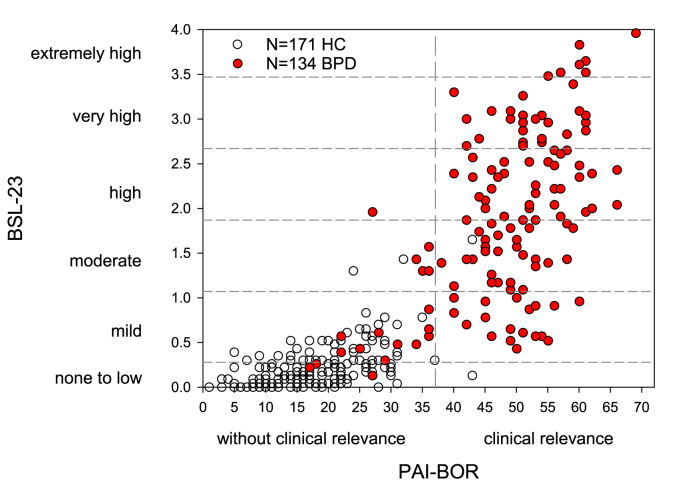
Scatterplot of PAI-BOR and BSL-23 scores across individuals diagnosed with BPD (filled circles) and non-clinical controls (HC), N = 305 Note: Each circle represents one individual with white circle for healthy controls (HC) and filled circles for individuals diagnosed with BPD during the last 2 years. The horizontal lines mark symptom severity categories of BPD for the BSL-23. The vertical line marks the cut-off between PAI-BOR scores corresponding to clinically non-significant and significant levels of Borderline personality features.

This short communication illustrates the issues we are currently facing for Borderline Personality Disorder research. Similar problems might arise in many research efforts that in the future aim to address personality, or symptoms transdiagnostically and across the entire population when investigating their biopsychosocial mechanism. These issue may be addressed through the integration of existing instruments with the employment of adaptive testing techniques, or a shift in the conceptualisation of disorders, for example, by describing and quantifying the psychopathology of personality disorders by maladaptive variants as extensions of personality traits that are already well-established in differential psychology (for example, (Skodol et al., 2017)). However, clarification of differences and similarities of the construct(s) associated with the dimensional and categorical models constitutes a prerequisite to align research findings and evaluate whether measurement instruments capture a dimensional attribute across the whole continuum ranging from varying intensities in healthy individuals up to variation in clinically relevant expressions in mental disorders with comparable reliability and validity (see e.g. (Bohnke and Croudace, 2015)). However so far against the backdrop of the - sometimes heated - debates in the context of the ‚replication crisis’, we suggest that differences between measurement instruments might be an under-appreciated reason for the failure to replicate research findings. Without addressing the described challenges, research findings may simply diverge depending on the measurement instrument used.

## Funding

Anna Schulze was supported by the 10.13039/501100001659Deutsche Forschungsgemeinschaft (DFG, German Research Foundation).

## Declaration of competing interest

The authors declare that they have no known competing financial interests or personal relationships that could have appeared to influence the work reported in this paper.
